# Improvement of hemodynamic parameters in aortic stenosis patients with transcatheter valve replacement by using impedance cardiography

**DOI:** 10.3389/fcvm.2022.950437

**Published:** 2022-09-20

**Authors:** Luqing Wan, Jianjun Tang, Yanchao Xiao, Hui Li, Zengjin Peng, Dan-Yan Xu, Li Shen

**Affiliations:** Department of Internal Cardiovascular Medicine, Second Xiangya Hospital, Central South University, Changsha, China

**Keywords:** aortic stenosis, transcatheter valve replacement, impedance cardiography, hemodynamic changes, cardiovascular diseases

## Abstract

**Background:**

The hemodynamic changes of patients with aortic stenosis (AS) who underwent transcatheter valve replacement (TAVR) have not been completely investigated.

**Methods and results:**

We enrolled 74 patients with AS who underwent TAVR and assessed cardiac function changes at 1 week post-operation by impedance cardiography (ICG) in a supine position at rest for more than 15 min. Of the 74 patients, 47 had preserved left ventricular ejection fraction (LVEF ≥ 50%; preserved-LVEF group) and 27 had reduced LVEF (LVEF <50%; reduced-LVEF group). TAVR improved the cardiac structure and function, as evidenced by the decrease in the left ventricular end-diastolic (LVED), left atrial diameter (LAD), and an increase in the LVEF. We observed a decrease in N-terminal pro-brain natriuretic peptide (NT-proBNP) level compared to that before treatment. Moreover, patients with reduced LVEF had a more significant reduction of NT-proBNP than those with preserved LVEF. Meanwhile, the blood pressure of patients had no significant differences pre- and post-operation. Based on ICG, there were no changes in the parameter of cardiac preload [thoracic fluid content (TFC)]. We observed an improvement in parameters of diastolic cardiac function [left ventricular ejection time (LVET) and pre-ejection period (PEP)]. And we detected converse results in parameters of heart systolic function [systolic time ratio (STR), cardiac output (CO), cardiac index (CI), stroke index (SI), and stroke volume (SV)] and cardiac afterload [stroke systemic vascular resistance (SSVR) and SSVR-index (SSVRI)]. In addition, TFC level was decreased in patients with thoracic volume overload after valve replacement. Subgroup analysis showed that the changes in those parameters were more noticeable in patients with reduced LVEF than that with preserved LVEF. Moreover, we observed no effects on parameters of heart systolic function and heart afterload in the LVEF ≥ 50% group before and after TAVR.

**Conclusion:**

Our data revealed a beneficial effect of TAVR in diastolic function and preload as detected by the ICG. But the LV systolic function and cardiac afterload were not improved in patients with LVEF <50%. The result indicated that ICG could be used as an important technique to monitor the cardiac condition of patients after aortic valve replacement.

## Introduction

Aortic stenosis (AS) is the most common valvular disease in developed countries. The incidence is 0.3–0.5% in the general population under 60 years of age and up to 2–7% in elderly people over 65 years of age (1). Due to the poor efficacy of medical treatment, patients should be considered for surgical intervention in presence of clinical symptoms, which increases the burden on medical systems and resources. In the early stage of AS, left ventricular pressure overload gradually increases left ventricular wall thickness, which causes left ventricular diastolic dysfunction. AS also accelerates the progression of impaired systolic function, leading to the decompensation stage of heart failure (HF).

After a long period of asymptomatic clinical latency, once patients with AS develop symptoms, the average life expectancy would reduce to 3 years (2). Considering the limited efficacy and survival rate of medical therapy, the surgical treatment for AS consisting of transcatheter aortic valve replacement (TAVR) and surgical aortic valve replacement (SAVR) has emerged as a viable option in patients with moderate or severe AS. The 2017 American College of Cardiology/American Heart Association (ACC/AHA) guidelines recommend performing TAVR in patients with high surgical risk for SAVR (3). Additionally, improved survival and surgical outcomes of TAVR demonstrated that it was equal to or superior to SAVR in low-surgical-risk patients according to a large clinical trial (4, 5).

Despite the rapid development of valve replacement technology, regular clinical and hemodynamics examinations are necessary to monitor the effects and consequences of implantation. Romeo et al. (6) reported that stroke volume/myocardial volume ratio ≤ 30% was associated with a poor prognosis after valve transplantation. Furthermore, the morbidity and mortality of 3-year major adverse cardiac or cerebrovascular events were reduced more in patients with immediate left ventricular ejection fraction (LVEF) improvement following TAVR than those without changes (7). Both studies suggested that cardiac hemodynamics and function were correlated positively to prognosis in AS patients receiving TAVR.

In the present study, the main method of hemodynamic monitoring was a cardiac ultrasound, which could evaluate cardiac structure and blood flow. Doppler ultrasound showed subtle changes in systolic function in the early stage of TAVR (8); however, it could not show the change in diastolic function immediately in AS patients after TAVR. The study by Spethmann et al. (9) reported that LV diastolic parameters, including the atrial fraction and left atrial volumes, had no significant improvements 8 days after surgery. Meanwhile, TAVR improved the LV structural remodeling at 6 months of follow-up (10) and reduced the percentage of patients with left ventricular dysfunction diseases from 65 to 49% at 1-year (11). These studies demonstrated that the improvement of cardiac structure is a long process and emphasized the necessity of monitoring the heart function of AS patients undergoing TAVR. However, expensive equipment, operator dependency, and complex data analysis limited the application of Doppler ultrasound.

Impedance cardiography (ICG) is a technique using electrodes to evaluate heart function, which is helpful for prognostication and diagnosis. The hemodynamics changed, responding to the changes in blood flow and volumes from beat to beat. Packer et al. (12) showed that ICG could predict clinical outcomes in stable HF patients. In their study, the score of a combination of three ICG parameters (velocity index, pleural fluid content index, and left ventricular ejection time) was a strong predictor of rehospitalization due to decompensated HF within 14 days (41.6% event rate of high-risk score vs. 1.0% event rate of a low-risk score, *P* < 0.001). Packer et al. (12) further found that thoracic fluid content (TFC) predicted the HF severity correctly, which showed high sensitivity and accuracy for volume overload. Moreover, ICG was used to check the valve conditions. According to the study described by Daralammouri et al. (13), there was a consistency between ICG and the Gorlin equation method, which was a gold standard for aortic valve size measurement (*r* = 0.76, *P* < 0.001). Another study reported that ICG monitored the restoration of cardiac function following coronary artery bypass grafting. They observed a decrease in the cardiac index and stroke volume index by non-invasive hemodynamic service (14). Moreover, ICG was an attractive tool for its non-invasive, low-cost, inexpensive, and simple to continuously monitoring cardiac function. However, the cardiac hemodynamic changes of AS patients who underwent TAVR are still unclear. Our study aimed to assess the effects of TAVR on cardiac function and hemodynamics non-invasively by ICG.

## Materials and methods

### Study population

The study was approved by the ethics committee of the Second Xiangya Hospital of Central South University, and written informed consent was obtained before its beginning. The study was conducted on a sample of 74 participants (27 women and 47 men) at the Second Xiangya Hospital of Central South University (Changsha, China) from 2020 to 2021. We collected information about the characteristics of the studied population before the surgery, including age, gender, height, weight, body mass index (BMI), heart rate (HR), blood pressure, and past medical history.

### Inclusion criteria

We enrolled patients with severe AS defined as (1) aortic velocity ≥ 4.0 m/s or mean transvalvular pressure gradient > 40 mmHg and (2) aortic valve area ≤ 1.0 cm^2^ or aortic valve area index <0.5 cm^2^/m^2^. For patients with low-flow, low-gradient severe AS, aortic velocity <4.0 m/s and mean transvalvular pressure gradient ≤ 40 mmHg were noted in electrocardiography. All participants had a clinical manifestation of AS with New York Heart Association (NYHA) class I to III.

### Exclusion criteria

The exclusion criteria were as follows: (1) uncontrolled arrhythmia; (2) acute pulmonary diseases, including acute exacerbation of asthma or chronic obstructive pulmonary disease, pneumothorax, and pleural effusion; (3) severe inflammation, infective endocarditis, pulmonary infarction, malignancies, hematological diseases, peripheral vascular diseases, and embolic diseases; (4) thyroid dysfunction and febrile; (5) other myocardiopathy; (6) history of cerebral hemorrhage, surgery, trauma, or glucocorticoid/immunosuppressive drug usage in the preceding 6 months; (7) aortic dissection; (8) muscle, nerve or mental illness; and (9) severe arrhythmia after TAVR.

### Biochemical and transthoracic echocardiogram measurements

All parameters were obtained 24 h before and 1 week after the surgical intervention. Venous blood was taken from an antecubital vein to measure the level of N-terminal pro-brain natriuretic peptide (NT-proBNP) after at least 8 h of fasting. NT-proBNP was measured by an Elecsys 2010 instrument (Roche Diagnostics) (15). The four-chamber image was saved by the Acuson SC2000 ultrasound system (Siemens Heathineers, Erlangen) with a 3–5 MHz transducer (16). Echocardiography was performed at the left lateral decubitus position of patients to assess their left atrium dimension (LAD), right atrial dimension (RAD), left ventricular end-diastolic (LVED), right ventricular dimension (RVD), and the LVEF. Measurements were obtained by an experienced independent cardiac sonographer. In addition, all laboratory and echocardiographic data were measured again using the same analyzer 1 week after TAVR.

### ICG measurements

As described in the previous clinical study, ICG was performed with a BioZ ICG device (CSM3000, QIANFAN electronics medical, Shenzhen, China) 24 h before and 1 week after the treatment (17). After a rest period of at least 15 min, the participants were in the supine position. All measurements of ICG were performed according to four pairs of sensors: two pairs applied bilaterally to the neck under each ear and two pairs to the thorax in the mid-axillary line at the level of the xiphoid. In the meantime, the cuff of an automated non-invasive blood pressure monitor was applied to the left arm of the patient. After the ICG waveform was stable on the ICG machine screen, the hemodynamic data were collected. Then, the operator printed and stored the ICG reports for analysis. The definition and calculation formulas of hemodynamic variables measured by ICG, including thoracic fluid content (TFC), pre-ejection period (PEP), left ventricular ejection time (LVET), systolic time ratio (STR), cardiac output (CO), cardiac index (CI), stroke index (SI), stroke volume (SV), stroke systemic vascular resistance (SSVR), and SSVR-index (SSVRI), were shown in Table 1 (18–20).

**Table 1:** Parameters assessed in impedance cardiography.

**Parameter**	**Definition**	**Formula**
**Diastolic function**		
Pre-ejection period (PEP)	The time between the electrical activation of the ventricles and the opening of the aortic valve	The time between electrical systole (ICG Q wave) to the initial opening of the aortic valve
Systolic time ratio (STR)	The ratio of the pre-ejection period divided by the left ventricular ejection time	PEP/LVET
Left ventricular ejection time (LVET)	The time from the aortic valve opening to the aortic valve closing	The time difference between points B and X of dZ/dt wave
**Systolic function**		
Cardiac output (CO)	The volume of blood expelled by the heart in 1 min	SV * HR
Stoke volume (SV)	Quantity of blood ejected from the cardiac ventricles for each heartbeat	ρ * L^2^/${\text{Z}}_{0}^{2}$ * dZ/dt_max_ * LVET
Cardiac index (CI)	Standard CO for the BSA	CO/BSA
Stroke index (SI)	Standard SV for the BSA	SV/BSA
**Fluid status**		
Thoracic fluid content (TFC)	The fluid content of the thorax, including intravascular, interalveolar and interstitial	The reciprocal of the total thoracic impedance
**Vascular resistance**		
Stroke systemic vascular resistance (SSVR)	Mean arterial blood pressure/CO	[80 * (MAP – CVP)]/CO
SSVR-index (SSVRI)	Standard SSVR for the BSA	SSVR/BSA

HR, heart rate; ρ, resistivity of blood; L, distance between the voltage measuring electrodes; Z0, basic thoracic impedance; dZ/dtmax, maximum impedance change; BSA, body surface area; MAP, mean artery pressure; CVP, central venous pressure (estimated value of 10 mmHg).

### Statistical analysis

All analysis was performed with IBM SPSS version 25, and statistical significance was set as a two-tailed *P* < 0.05. Continuous data were presented as mean ± standard deviation while categorical data were presented as number and %. We used the chi-square tests (or Fisher exact tests as appropriate) to compare categorical variables and paired student's *t*-tests for continuous variables. In addition, the paired Kruskal–Wallis tests or paired Wilcoxon signed-rank tests were used when samples existed outside the normal distribution.

## Results

### Baseline patient characteristics

A total of 74 patients were enrolled in this study, and 47 of them were male. The general characteristics of the study population are shown in Table 2. The mean age was 68 ± 10 years, the height was 1.61 ± 0.08 m, the weight was 59.51 ± 11.66 kg, and the BMI was 22.91 ± 3.64 kg/m^2^. In this study, hypertension, diabetes, and coronary heart disease were found in 25 (34%), 15 (20%), and 27 (36%) patients, respectively. For a comparative analysis, the participants were divided into two subgroups: patients with LVEF ≥ 50% (*n* = 47) and LVEF <50% (*n* = 27).

**Table 2:** The baseline characteristics between patients with LVEF <50% and LVEF ≥ 50%.

**Characteristic**	**All patients**	**LVEF <50% patients**	**LVEF ≥50% patients**
	***n* =74**	***n* = 27**	***n* = 47**
Age (years)	67.96 ± 9.76	67.93 ± 9.74	67.98 ± 9.88
Gender (male, %)	47 (63.5)	17 (23.0)	30 (40.5)
Height (m)	1.61 ± 0.08	1.61 ± 0.07	1.61 ± 0.08
Weight (kg)	59.51 ± 11.66	58.89 ± 12.89	59.87 ± 11.02
BMI (kg/m^2^)	22.91 ± 3.64	22.56 ± 3.78	23.11 ± 3.59
Hypertension (*n*, %)	25 (33.8)	6 (8.1)	19 (25.7)
Diabetes (*n*, %)	15 (20.3)	6 (8.1)	9 (12.2)
CHD (*n*, %)	27 (36.5)	8 (10.8)	19 (25.7)

Continuous variables are mean ± SD. All other variables are numbers and percentages.

BMI, body mass index; CHD, coronary heart disease.

### Comparison of transthoracic echocardiogram pre- and post-TAVR

The echocardiographic results of RVD and RAD did not differ between the pre- and post-operative periods (Table 3). In comparison, LVED and LAD decreased (54.21 ± 8.67 vs. 51.00 ± 7.49 mm; 40.48 ± 6.71 vs. 38.06 ± 6.09 mm, both *P* < 0.05), and LVEF increased significantly (51.69 ± 11.93 vs. 54.48 ± 12.15%, *P* < 0.05). In both subgroups, comparing data recorded at 1 week of treatment with TAVR to those recorded at baseline, we found the same change in RVD, RAD, LVED, and LAD. Furthermore, patients in LVEF <50% group experienced a more significant increase in LVEF than that in the LVEF ≥ 50% group (reduced-LVEF group, 38.04 ± 6.11 vs. 43.58 ± 10.84%, *P* < 0.05; preserved-LVEF group, 59.58 ± 5.60 vs. 60.78 ± 7.57%, *P* > 0.05; Table 3).

**Table 3:** Echocardiographic data at baseline and the end of the study.

		**LVED (mm)**	**LAD (mm)**	**RVD (mm)**	**RAD (mm)**	**LVEF (%)**
All patients	Pre-TAVR	54.21 ± 8.67	40.48 ± 6.71	31.94 ± 3.81	33.97 ± 5.06	51.69 ± 11.93
	Post-TAVR	51.00 ± 7.49^***^	38.06 ± 6.09^***^	31.97 ± 3.99	33.96 ± 4.86	54.48 ± 12.15^*^
LVEF <50% group	Pre-TAVR	61.56 ± 7.73	44.52 ± 6.99	34.00 ± 4.27	36.77 ± 6.09	38.04 ± 6.11
	Post-TAVR	57.16 ± 7.44^**^	38.76 ± 6.18^***^	33.38 ± 4.26	36.31 ± 5.56	43.58 ± 10.84^*^
LVEF ≥ 50% group	Pre-TAVR	50.13 ± 6.13	38.28 ± 5.47	30.78 ± 3.00	32.32 ± 3.45	59.58 ± 5.60
	Post-TAVR	47.58 ± 4.92^**^	37.46 ± 5.95^*^	31.17 ± 3.64	32.57 ± 3.82	60.78 ± 7.57
*P*-value		<0.001	<0.001	0.930	0.970	0.010
P^*^-value		0.002	<0.001	0.206	0.340	0.010
*P^#^*-value		0.005	0.029	0.363	0.600	0.228

Continuous variables are mean ± SD.

LVED, left ventricular end-diastolic; LAD, left atrium dimension; RVD, right ventricular dimension; RAD, right atrium dimension; LVEF, left ventricular ejection fraction.

P-value of comparison between changes in all patients before and after TAVR.

P^*^-value of comparison between changes in LVEF <50% group before and after TAVR.

P^#^-value of comparison between changes in LVEF ≥ 50% group before and after TAVR.

* P-value <0.05,

** P-value <0.01, and

*** P-value <0.001 when compared to baseline.

### The comparison of NT-proBNP levels at baseline and following treatment

Table 4 shows the level of NT-proBNP. We observed a significant reduction of NT-proBNP compared to that before treatment (*P* < 0.001). After 1 week of TAVR, a significant reduction was detected in both groups (all *P* < 0.05). The decrease of NT-proBNP in patients with reduced LVEF was larger than that in patients with preserved LVEF (5,081 ± 6,332 vs. 624 ± 1,634 pg/ml, *P* < 0.001).

**Table 4:** The comparison of NT-proBNP at baseline and the end of the study.

**Variable**	**All patients**		**LVEF**<**50% group**		**LVEF** ≥**50% group**		***P*-value**	P^*^-value	***P^#^-*value**
	**Pre-TAVR**	**Post-TAVR**	**Pre-TAVR**	**Post-TAVR**	**Pre-TAVR**	**Post-TAVR**			
NT-proBNP	6,749 (899, 8,419)	3,129 (609, 2,522)^***^	14,203 (3,073, 19,146)	6,150 (1,634, 6,838)^***^	1,903 (435, 2,543)	1,164 (446, 1,384)^*^	<0.001	<0.001	0.030

Continuous variables are mean (25th percentile, 75th percentile).

NT-proBNP, N-terminal brain-type natriuretic peptide.

P-value of comparison between changes in all patients before and after TAVR.

P^*^-value of comparison between changes in LVEF <50% group before and after TAVR.

P^#^-value of comparison between changes in LVEF ≥ 50% group before and after TAVR.

* P-value < 0.05 and

*** P-value < 0.001 when compared to baseline.

### Comparison of blood pressure in all patients before and after TAVR 1 week

As shown in Table 5, there was no difference in systolic blood pressure (SBP) and diastolic blood pressure (DBP) after TAVR. Additionally, the difference in blood pressure post-surgery in the two subgroups was not obvious.

**Table 5:** The comparison of SBP and DBP at baseline and the end of the study.

**Variable**	**All patients**		**LVEF**<**50% group**		**LVEF** ≥**50% group**		***P-*value**	P^*^-value	***P^#^-*value**
	**Pre-TAVR**	**Post-TAVR**	**Pre-TAVR**	**Post-TAVR**	**Pre-TAVR**	**Post-TAVR**			
SBP (mmHg)	114.26 ± 20.38	110.57 ± 15.99	107.08 ± 20.43	106.27 ± 15.56	118.5 ± 19.35	113.11 ± 15.87	0.136	0.846	0.084
DBP (mmHg)	62.73 ± 13.03	62.36 ± 9.51	60.96 ± 12.95	61.67 ± 7.93	63.65 ± 13.11	62.72 ± 10.30	0.842	0.792	0.708

Continuous variables are mean ± SD.

SBP, systolic blood pressure; DBP, diastolic blood pressure.

P-value of comparison between changes in all patients before and after TAVR.

P^*^-value of comparison between changes in LVEF <50% group before and after TAVR.

P^#^-value of comparison between changes in LVEF ≥ 50% group before and after TAVR.

### Changes in parameters of ICG after TAVR

#### Cardiac preload

Cardiac function was evaluated by ICG at baseline and the end of 1 week after TAVR. No difference was found in the level of TFC (0.030 ± 0.006 vs. 0.030 ± 0.005 1/Ω, *P* > 0.05; Table 6). After sub-dividing subjects into high-TFC group (TFC > 0.035 1/Ω) and normal-TFC group (TFC ≤ 0.035 1/Ω), the subgroup analysis indicated a different result. The TFC increased in patients with TFC ≤ 0.035 1/Ω (0.027 ± 0.004 vs. 0.029 ± 0.004 1/Ω, *P* < 0.05), whereas TFC decreased in patients with TFC > 0.035 1/Ω post-TAVR (0.039 ± 0.005 vs. 0.035 ± 0.006 1/Ω, *P* < 0.05).

**Table 6:** Fluid content in all patients and subgroup patients divided by TFC = 0.035 1/Ω before and after TAVR.

**Variable**	**All patients**		**TFC** ≤ **0.035 group**		**TFC** > **0.035 group**		***P*-value**	P^*^-value	***P^#^*-value**
	**Pre-TAVR**	**Post-TAVR**	**Pre-TAVR**	**Post-TAVR**	**Pre-TAVR**	**Post-TAVR**			
TFC (1/Ω)	0.030 ± 0.006	0.030 ± 0.005	0.027 ± 0.004	0.029 ± 0.004*	0.039 ± 0.005	0.035 ± 0.006*	0.391	0.020	0.032

Continuous variables are mean ± SD.

TFC, thoracic fluid content.

P-value of comparison between changes in all patients before and after TAVR.

P^*^-value of comparison between changes in TFC ≤ 0.035 1/Ω group before and after TAVR.

P^#^-value of comparison between changes in TFC > 0.035 1/Ω group before and after TAVR.

#### Diastolic function of heart

Our results revealed that TAVR improved the diastolic heart function as reflected by the decrease of LVET (303.99 ± 39.41 vs. 264.01 ± 40.79 ms, *P* < 0.001; Table 7) and increase of PEP (107.71 ± 21.66 vs. 118.04 ± 27.04 ms, *P* < 0.05). Subgroups analysis indicated that TAVR also improved the diastolic heart function in both groups, including a decrease in LVET and an increase in PEP (both *P* < 0.05).

**Table 7:** Diastolic function, systolic function, and circulation function in all patients and subgroups before and after TAVR.

		**PEP (ms)**	**LVET (ms)**	**STR**	**CO (L/min)**	**CI (L/min/m^2^)**	**SI (mL/m^2^)**	**SV (mL)**	**SSVR (dynes/cm^5^/beats)**	**SSVRI (dynes/cm^5^/beats/m^2^)**
All patients	Pre-TAVR	107.71 ± 21.66	303.99 ± 39.41	0.36 ± 0.10	4.55 ± 1.29	2.96 ± 0.70	41.97 ± 10.53	66.91 ± 18.56	249.17 ± 78.09	143.73 ± 43.61
	Post-TAVR	118.04 ± 27.04^**^	264.01 ± 40.79^***^	0.47 ± 0.13^***^	4.16 ± 0.97^***^	2.66 ± 0.62^***^	38.17 ± 9.28^***^	61.64 ± 15.19^**^	269.93 ± 73.33^**^	173.64 ± 60.95^***^
LVEF <50% group	Pre-TAVR	112.85 ± 23.64	309.25 ± 36.80	0.38 ± 0.10	4.29 ± 1.49	2.94 ± 0.70	39.36 ± 10.13	64.00 ± 19.93	230.60 ± 57.86	135.56 ± 29.14
	Post-TAVR	126.88 ± 30.66^*^	275.21 ± 28.44^***^	0.52 ± 0.13^***^	3.68 ± 0.92^**^	2.40 ± 0.45^***^	33.88 ± 6.26^**^	55.28 ± 11.60^**^	272.40 ± 58.66^*^	198.30 ± 65.21^***^
LVEF ≥ 50% group	Pre-TAVR	104.6 ± 20.01	301.18 ± 40.86	0.35 ± 0.09	4.70 ± 1.14	2.97 ± 0.70	43.36 ± 10.58	68.53 ± 17.78	258.46 ± 85.58	147.63 ± 48.86
	Post-TAVR	112.70 ± 23.38^*^	258.04 ± 45.21^***^	0.44 ± 0.12^***^	4.45 ± 0.89	2.80 ± 0.66	40.45 ± 9.86	65.18 ± 15.89	268.70 ± 80.30	161.85 ± 55.75
*P*-value		<0.001	<0.001	0.003	<0.001	<0.001	<0.001	0.002	0.047	<0.001
P^*^-value		<0.001	<0.001	0.038	0.008	<0.001	0.004	0.005	0.023	<0.001
*P^#^*-value		<0.001	<0.001	0.034	0.065	0.144	0.055	0.093	0.421	0.112

Continuous variables are mean ± SD.

PEP, pre-ejection period; STR, systolic time ratio; LVET, left ventricular ejection time; CO, cardiac output; CI, cardiac index; SI, stroke index; SV, stroke volume; SSVR, stoke systemic vascular resistance; SSVRI, stroke systemic vascular resistance index.

P-value of comparison between changes in all patients before and after TAVR.

P^*^-value of comparison between changes in TFC ≤ 0.035 group before and after TAVR.

P^#^-value of comparison between changes in TFC > 0.035 group before and after TAVR.

* P-value < 0.05,

** P-value < 0.01, and

*** P-value < 0.001 when compared to baseline.

#### Systolic function of the heart

After TAVR, the systolic function was not improved as reflected by the increase of STR (0.36 ± 0.10 vs. 0.47 ± 0.13, P <0.001) and the decrease of CO (4.55 ± 1.29 vs. 4.16 ± 0.97 L/min, *P* < 0.05; Table 7), CI (2.96 ± 0.70 vs. 2.66 ± 0.62 L/min/m^2^, *P* < 0.05), SI (41.97 ± 10.53 vs. 38.17 ± 9.28 mL, *P* < 0.05), and SV (66.91 ± 18.56 vs. 61.64 ± 15.19 mL/m^2^, *P* < 0.05). In the subgroup analysis, the changes of CO, CI, SI, and SV were not significant in the preserved-LVEF group (all *P* > 0.05), while the levels of those parameters were decreased in the reduced-LVEF group after implanting the artificial valve (all *P* < 0.05). The level of STR was increased in both groups.

#### Cardiac afterload

In addition, SSVR and SSVRI increased (SSVR, 249.17 ± 78.09 vs. 269.93 ± 73.33 dynes/cm^5^/beat; SSVRI, 143.73 ± 43.61 vs. 173.64 ± 60.95 dynes/cm^5^/m^2^/beat, both *P* < 0.05; Table 7) in post-TAVR patients. After the subgroups dividing, the levels of SSVR and SSVRI were increased in patients with LVEF <50%. However, there were no statistically significant differences in the level of those parameters in patients with LVEF ≥ 50% post-TAVR.

## Discussion

After valve replacement, echocardiographic data showed lower LVED and LAD and higher levels of LVEF, and the differences were more pronounced in patients with LVEF <50%. The NT-proBNP showed a decrease in all patients, and the reduction in the LVEF <50% group was also much greater than that in the LVEF ≥ 50% group. The SBP and DBP remained unchanged throughout the study period. In this study, we assessed hemodynamic parameters by ICG in AS patients who underwent TAVR. Our results showed that TAVR decreased the level of TFC in those with thoracic volume overload. Additionally, the ICG parameters of heart diastolic function showed an immediate improvement compared to those before TAVR. Meanwhile, there was a decrease in CO, CI, SI, and SV and an increase in STR, SSVR, and SSVRI in patients with reduced LVEF, which indicated that the cardiac systolic function and the cardiac afterload were not improved in poorer LVEF patients. At the same time, TAVR did not change the parameters of CO, CI, SI, SV, SSVR, and SSVRI in patients with preserved LVEF.

Previously published studies have shown that the diagnostic criteria for patients with severe AS are aortic valve pressure gradient ≥ 40 mmHg and aortic valve area <1.00 cm^2^. Following TAVR, the aortic valve pressure gradient and aortic valve area reduced (21), while the LVEF increased (39.3 ± 8.8 vs. 44.1 ± 10.1%, *P* < 0.05), which indicated that TAVR improved the cardiac function. Our data suggested that LAD and LVED reduced, LVEF increased, and RAD and RVD did not change after TAVR. These findings could be explained by the removing obstruction of the aortic valve area by TAVR, which reduced the load and systolic pressure of the LA and LV. As a result, LAD and LVED decreased, and LVEF increased. Jeong et al. (7) showed that the LVEF improved immediately after TAVR, and the improvements occurred mostly in patients with LVEF ≤ 55% within 30 days after surgery, which was consistent with our study. In addition, we showed that patients with reduced LVEF had a significant increase in LVEF at 1 week of TAVR. However, the changes in the group of LVEF ≥ 50% had no significant differences. It indicated that the level of baseline LVEF was correlated with the improvement of LV systolic function.

The recovery of cardiac function in patients after TAVR is closely related to prognosis, evidenced by a decreased rate of rehospitalization from HF in patients with LVEF improvement ≥ 10% undergoing TAVR (22). Another study showed that the BNP level fell sharply (23), which was positively correlated with the risk of cardiovascular death and all-cause mortality at 2 years after TAVR. Our data showed that the level of NT-proBNP reduced statistically after surgery. After dividing the patients into two subgroups based on LVEF <50% and LVEF ≥ 50%, we found that the NT-proBNP decreased more significantly in the reduced-LVEF group. Moreover, Sherif et al. (24) found that the improvement of HF after TAVR in the EF <40% group was more significant. The difference could be explained by two reasons, one was that the left ventricular systolic function recovered more fully in the LVEF <50% group and another was that the severity of HF of the patients in the LVEF ≥ 50% group was low, so the reduction of NT-proBNP after TAVR was more significant in the LVEF <50% group.

The results of blood pressure in our study showed that the SBP and DBP remained unchanged after TAVR. Subgroups analysis showed a similar result, which indicated that BP changed little after TAVR. Niu et al. (25) observed an increase of SBP in most patients at 30 days of TAVR, and it was possible to hypothesize that the detection time may be too short to observe the BP changes.

ICG uses the thoracic electrical impedance to assess hemodynamics and possesses good prospects in clinical application. A study showed that after coronary artery bypass graft, the EF, SV, CO, and CI monitored by ICG were correlated with the results obtained by echocardiography (26). TFC is an indicator of ICG that reflects the amount of fluid in the chest cavity, which could reflect the cardiac preload by recording the changes in fluid impedance. The study by Nazzari et al. (27) showed that patients after TAVR had an increased risk of rehospitalization due to HF progression (20% within 30 days vs. 59.7% within 1 year), and the mortality of patients with HF was higher than that without HF (25% vs. 10.9%, *P* < 0.001). It was obvious that HF was a postoperative complication, and there was particular importance in identifying HF and classifying the severity of HF patients through TFC. However, our study found that there was no statistical difference in the level of TFC, whereas the level of pro-BNP decreased significantly in patients undergoing TAVR. To explore the relationship between TFC and BNP, based on the results that patients with TFC > 0.035 1/Ω had more severe clinical symptoms of dyspnea or edema reported by Pimenta et al. (28), we divided patients into the TFC ≤ 0.035 and TFC > 0.035 1/Ω groups. Subsequent data analysis showed that TFC increased statistically in the TFC ≤ 0.035 1/Ω group, while decreased in the TFC > 0.035 1/Ω group (both *P* < 0.05). The changes in high TFC patients were consistent with the alternations in NT-proBNP, which supported that TFC was related to NT-proBNP in patients with thoracic volume overload. Consistent with our results, Balak et al. (29) found that in patients with stable hemodynamics and symptoms of HF, there was no significant correlation between TFC and BNP levels. At the same time, there was a strong positive correlation between TFC and BNP in patients with serious HF.

PEP and LVET had been used to describe cardiac contractility since ICG was applied; however, a recent study (30) verified that those parameters were identified as good left ventricular diastolic dysfunction screening tools with high sensitivity and specificity in hypertensive patients (> 90%). They observed an increase of LVET and a shortening of PEP in patients with hypertension. Hypertension had a similar pathological mechanism to AS, which increased vascular load and impaired cardiac diastolic function in the early stage of diseases. Considering that it was also the most common comorbidity (incidence rate = 78%) in old patients with AS and an independent risk factor of AS (31, 32), we assumed that PEP and LVET could reflect the cardiac diastolic function of AS patients. Moreover, Weber et al. (33) showed that patients with diastolic dysfunction had longer LVETI, and LVETI could be used as an independent predictive factor for diastolic dysfunction. The results in our study were consistent with those studies, including the decrease of LVET and the increase of PEP, which supported the improvement of diastolic function in patients after TAVR. The subgroups analysis showed a more statistically significant difference in the LVEF <50% group than that in the LVEF-preserved group, which further verified our hypothesis. Geraldine Ong et al. (4) indicated that when the diastolic function improved within 30 days in patients after TAVR, the cardiovascular death and rehospitalization rates were lower at 1 year after the operation, and the survival rate was higher at 2 years, suggesting that the improved diastolic function after TAVR was related to the prognosis. Sang Jin Ha et al. (34) reported that the reduction of cardiac diastolic function was observed in almost 42% of patients after TAVR. Overall, these results provided important insights into diastolic function changes which may demonstrate the benefits of ICG.

STR, CO, CI, SI, and SV are usually used to reflect the cardiac output. Prior research had found that the parameters were associated with discharge time and the incidence of comorbidity following TAVR (35–37). We found that the CO, CI, SI, and SV decreased, and STR increased in the postoperative patients, suggesting that the myocardial contraction was impaired 1 week after TAVR. Subgroups analysis showed that the cardiac output of both groups was decreased; however, the changes were not significant in the LVEF ≥ 50% group. This is contrary to the study of Chrissoheris et al. (38). Their results showed that the LVEF improved in patients within 24 h after TAVR, and SV and CI increased. However, some mechanisms might be possible to explain the decrease in CO, CI, SI, and SV. Lewicki et al. (39) demonstrated that the potential myocardial damage in percutaneous coronary intervention (PCI) procedures decreased myocardial contractility and increased SSVR monitored by non-invasive hemodynamics. In addition, the results of Stundl et al. (40) showed that 51.6% of the patients after TAVR increased high-sensitivity troponin (hs-TnI), suggesting that there was a possibility of myocardial injury after TAVR. And the study of Kobayashi et al. (41) suggested that the increase in hs-TnI levels after TAVR is correlated with LVEF. Based on the results, we put forward a hypothesis that patients who underwent TAVR had impaired myocardial contractility. Our study supported these results, with the differences in STR, CO, CI, SI, and SV being absent in LVEF ≥ 50% group. Another explanation might be that the benefits in AS patients from TAVR were accumulated in a long-term way. Markus et al. (42) found a decreased CO and CI 6–8 h after TAVR; however, the parameters increased when comparing discharge to baseline. Moreover, all ICG parameters were derived from the change of the thoracic impedance during the cardiac cycle, and the reduced body fluid might mask the effects of cardiac function after TAVR (43).

SSVR is an index reflecting peripheral resistance. And SSVRI is the result of SSVR standardized by BSA (SSVR/BSA). Eliminating the interference of height and weight, SSVRI could more accurately represent the cardiac afterload. From a previous study, SSVR was positively associated with the severity of AS (44). In our data, SSVR and SSVRI both increased at a statistically significant level; however, the increase was not obvious in the LVEF ≥ 50% group by subgroups analysis. Indeed, Barbetseas et al. (45) showed an increased aortic stiffness after 1 week of SVR because of impaired vasa vasorum and aortic wall. They compared the values of aortic root function indices, such as aortic stiffness index and aortic root distensibility, and indicated that the aortic wall was impaired due to the operative procedure of TAVR. Moreover, another study stated by Yotti et al. (46) suggested the aortic valve stiffness exhibited in AS patients. They showed that long-time valve obstruction increased vascular load and valve wall stiffness, and patients after TAVR exhibited stiffer aortic valve behavior evidenced by the increase of SVRI. In their study, the increased vascular load correlated with the decrease of stroke volume index (a parameter of LV systolic function). Our results supported the hypothesis that the increase of SSVR and SSVRI post-procedure and the changes were smaller in the preserved-LVEF group than that in the reduced-LVEF group.

Using ICG to estimate hemodynamic parameters, an improvement could be noted to some extent in the heart diastolic function with AS patients who underwent TAVR, but we acknowledge the echocardiographic parameters of diastolic cardiac function, including e', E/e', E/A, and DT (Deceleration time), should be evaluated. If patients with reduced LVEF had an improvement in diastolic function assessed by echocardiography after TAVR, it would be consistent with our data, which could provide higher reliability of ICG. It is important to extend follow-up time, enlarge the sample size, and add specific diastolic function measurements in further study.

We found that TAVR reduced chest fluid and improved diastolic function monitored by ICG. However, the systolic function and the afterload were not improved in patients after 1 week of TAVR. Our results suggested that non-invasive hemodynamic monitoring could assess cardiac function and provide timely treatment that would improve the prognosis and outcomes of AS patients following TAVR.

## Data availability statement

The original contributions presented in the study are included in the article/supplementary material, further inquiries can be directed to the corresponding author.

## Ethics statement

The studies involving human participants were reviewed and approved by the National Nature Scientific Funding of China (No. 82072555). The patients/participants provided their written informed consent to participate in this study.

## Author contributions

LW, LS, and JT contributed to conception and design of the study. LW organized the database and wrote the first draft of the manuscript. YX performed the statistical analysis. HL, ZP, D-YX, and LS wrote sections of the manuscript. All authors contributed to manuscript revision, read, and approved the submitted version.

## Funding

This work was supported by the National Nature Scientific Funding of China (No. 82072555), Chinese Cardiovascular Association-Access fund (2019-CCA-ACCESS-023), and the National Nature Scientific Funding of Hunan Province and Changsha (2021JJ30948, 202203013201).

## Conflict of interest

The authors declare that the research was conducted in the absence of any commercial or financial relationships that could be construed as a potential conflict of interest.

## Publisher's note

All claims expressed in this article are solely those of the authors and do not necessarily represent those of their affiliated organizations, or those of the publisher, the editors and the reviewers. Any product that may be evaluated in this article, or claim that may be made by its manufacturer, is not guaranteed or endorsed by the publisher.
